# Taxation of sugar sweetened beverages and unhealthy foods: a qualitative study of key opinion leaders’ views

**DOI:** 10.1186/s13584-018-0240-1

**Published:** 2018-07-31

**Authors:** Orly Tamir, Tamar Cohen-Yogev, Sharon Furman-Assaf, Ronit Endevelt

**Affiliations:** 1 The Gertner Institute for Epidemiology and Health Policy Research, Sheba Medical Center, Ramat Gan, Israel; 2 School of Public Health, Haifa University, Haifa, Israel

**Keywords:** Sugar sweetened beverages, Unhealthy snacks, Obesity prevention strategy, Taxation, Tax revenue

## Abstract

**Background:**

Fiscal policies to fight obesity such as taxation of unhealthy foods or sugar-sweetened beverages (SSBs) have gained considerable attention in recent years. Many studies modelling the impact of various magnitudes of taxes on SSB purchasing and their potential effects on various health outcomes have been published; however, legislation and implementation of such taxes have encountered many obstacles in the countries that have implemented them to date. We investigated the perceptions and views of key opinion leaders, policy makers and various other Israeli stakeholders on taxation of SSBs and unhealthy snacks. We also evaluated the challenges and barriers that may be expected for initiating such a policy.

**Methods:**

A qualitative study based on 39 in-depth interviews with Israeli stakeholders in the fields of health, nutrition, economics, public advocacy and policymaking.

**Results:**

All stakeholders viewed obesity as a combined societal and personal issue that should be under government responsibility. Only stakeholders from economic sectors thought that taxation of SSBs and unhealthy snacks would reduce their consumption, while the prevailing notion among non-economists was that such a tax would not be acceptable because the higher price would not decrease consumption. Concerns were raised that the tax would mostly affect individuals from low socioeconomic backgrounds. Some of the stakeholders indicated that they would support such a tax only if its revenue would be directed to specific causes such as health-promoting plans. Potential barriers to taxation include: opposition of various sectors, technical and bureaucratic obstacles impeding tax implementation, difficulties in defining which products to tax, and opposition of the treasury to earmark tax revenue for health education.

**Conclusions:**

Taxation should be a part of a multipronged strategy rather than a sole measure for fighting obesity. Dedicating tax revenues to specific predefined causes should be considered, particularly towards health promotion activities, obesity treatment and prevention, education, and subsidies of healthy food.

## Background

Worldwide obesity has more than doubled since 1980, reaching alarming proportions in developed countries [1, 2]. Poor nutrition and lack of physical activity have been identified as the main predictors of obesity [3]. Increased consumption of sugar-sweetened beverages (SSBs) among children and adolescents has been associated with higher caloric intake [4–7], as well as intake of ultra-processed foods [8]. Similar to the global pattern, obesity has increased in the Israeli population. In 2015, 25% of the population of Israel was estimated to be obese and an additional 30% were overweight. Among Israeli 15 year-olds, 31% of girls and 33% of boys reported drinking SSBs daily in 2014 [9].

The idea of taxation as a public health intervention to reduce the consumption of products that have negative externalities dates back to 1776, when the Scottish philosopher and economist Adam Smith in his work “An Inquiry into the Nature and Causes of the Wealth of Nations” supported the taxation of tobacco, rum, and sugar by reasoning that none were considered necessary for life [10]. While taxation of tobacco for fiscal and health purposes is now universally accepted, taxation of unhealthy foods or SSBs to fight obesity has only gained considerable attention in recent years. In the last decade, an increasing number of countries have either implemented or proposed plans to introduce taxes on SSBs or on foods high in salt, fat and/or sugar. These include: Mexico [11, 12], France, Hungary, Finland, Latvia [13] Barbados, Belgium, Brunei Darussalam, Chile, Kiribati, Norway, Samoa, Saudi Arabia, Spain, Vanuatu, Mauritius, French Polynesia [14], Fiji and Tonga, Samoa, Nauru [15], South Africa [16] and the United Kingdom [17]. Many studies modelling the impact of various magnitudes of taxes on SSB purchasing and their potential effects on various health outcomes have been published. For example, it was estimated that taxation of SSBs has the potential to reduce the calorie-intake by more than 5000 kcal per capita [3–6]. Studies in Germany have suggested that implementing a 20% sales tax on SSBs is likely to have significant impact on overweight and obesity and reduce caries especially in young low-income males [18, 19]. Other studies have found a small but significant impact of such measures, depending on the type of food, and the type and level of the taxing scheme [20–22]. Despite modelling studies that show that increased price due to taxation will reduce consumption, the decisions leading to introducing a taxation policy on unhealthy foods depend on the interplay and influence of various interest groups, including government agencies, food industry lobbyists, public health professionals, consumer groups and public representatives, on the legislators [23]. The viewpoints of various stakeholders representing these interest groups have not been thoroughly investigated to date.

In this study we examined the perceptions and views of key opinion leaders, policy makers and various other Israeli stakeholders on taxation of SSBs and unhealthy snacks. Specifically, we aimed to understand stakeholders’ views on the responsibility for tackling obesity; the use of negative incentives for health promotion; the applicability and acceptance of using taxation on SSBs and unhealthy snacks in Israel; and the possibility of earmarking taxes for specific causes. We also evaluated the challenges and barriers that may be expected for initiating such a policy.

## Methods

### Study design and interview guide

This qualitative study used a semi-structured interview guide constructed by the study team based on current literature [24–26] with the help of an expert on health promotion strategies. The topics of the interview are shown in Table 1.

**Table 1:** ᅟ

Interview stances	
1. According to your world-view, is obesity a personal problem or is it a problem/phenomenon that is of public interest? 2. In your opinion, who holds the responsibility for dealing (preventing and treating) with this issue? 3. What is your opinion on the issue of using negative incentives (i.e., increased taxation) for changing an individual’s lifestyle? 4. Certain countries have initiatives for taxation of unhealthy beverages and food. For example, in May 2010 in Washington DC, a 6% sales tax intended for funding of a physical activity program was imposed on sweetened beverages and energy drinks. What is your opinion about a similar initiative in Israel? (Would you be for it or would you oppose it and why, in terms of the organization that you represent). 5. In your opinion what would be the reaction of the public to such an initiative? (Please specify the reasons for your support or opposition). 6. Do you think that imposing such a tax would affect the public’s consumption of these products? 7. In your opinion, which sectors (apart from the general public) would be expected to oppose such tax? 8. What would be the reasons for opposing such tax? 9. What additional obstacles to implementation of this tax would be expected in Israel? 10. Who, in your opinion would support such a tax? 11. What is your opinion on such a tax if its revenues would be earmarked to specific causes (as compared to a general tax whose revenues are directed to the general treasury)? 12. To which causes should the tax’s revenues be directed?	

### Data collection

Data was collected between September 2011 and February 2012 through face-to-face interviews by two experienced interviewers with a background in health research methods and interviewing skills training. One of the interviewers conducted the interview and the other took notes. All interviews were recorded and transcribed. The interviewers kept field notes and each interview was debriefed immediately afterwards.

### Participants

Purposive sampling was used to obtain a sample of participants who possessed diverse characteristics and points of view that were likely to provide abundant information towards the subject under investigation. This method involves identifying and selecting individuals or groups of individuals that are especially knowledgeable about, or experienced with, a phenomenon of interest [27, 28]. The study team held brainstorming meetings together with experts from the health promotion field during which the various sectors whose representatives would be interviewed were suggested. The eight sectors chosen had the potential for providing representative information on various aspects that are relevant to the study question. To elicit insights from the diverse range of relevant stakeholders in each sector, persons considered highly experienced leaders in their respective sector were suggested as interviewees and subsequently approached. Of 56 potential interviewees who were approached, 39 (11 women and 28 men) agreed to participate in the study and 17 declined due to various reasons, mainly unavailability at the time of the study. Table 2 summarizes the participants’ characteristics.

**Table 2:** Characteristics of interviewees

Sector	Stakeholders	Number	Gender (n)	Average seniority (years)
Health professions	• Health professionals • Representatives of professional associations in the fields of nutrition, healthcare and health promotion	9	Females: 6 Males: 3	11
Legislators	• Members of the Israeli parliament from various political parties	6	Females: 1 Males: 5	7
Policy makers	• Council members of municipal authorities • Israeli government bureaucrats	4	Females: 1 Males: 3	7
Regulators	• Senior officers from the Ministry of Health, the Ministry of Finance and the Israel Tax Authority	4	Females: 1 Males: 3	14
Food and beverage industry	• Senior managers of snack and beverage manufacturers • Managers of national supermarket chains • Catering Managers	5	Males: 5	12
Public representatives	• Leaders of consumer-advocate groups • Social activists	3	Females: 1 Males: 2	18
Media and publishing	• Health and health economy journalists • Representatives of advertising agencies engaged in marketing of snacks and beverages	4	Females: 1 Males: 3	10
Economists	• Health economists	4	Males: 4	18
Total		39	Females: 11 Males: 28	11

### Data analysis

Data analysis was based on thematic analysis [29]. The Interviews were recorded and transcribed and field notes were reviewed. Each transcript was reviewed and a line-by-line content analysis was used to identify major themes and quotes. Research team members met to discuss the identification of key themes. Themes were defined as specific content that was mentioned in more than three interviews in the same sector or if specifically identified as a core issue in relation to the overall content of the interview. Quotes from interviews were extracted to support theme identification. Next, the individual themes were pooled and arranged systematically to identify higher order categories. A small number of themes were dropped from the final analysis due to insufficient content overlap and power to stand alone as a separate theme and reflected sufficient data collection to achieve saturation.

## Results

Ten main themes were identified (Table 3).

**Table 3:** Main categories and themes in the study

Category	Themes
The responsibilities of individual and society for obesity, its causes and treatment	• Obesity is a public and private problem • Obesity is a financial (economic) problem
Negative positions about taxation of sugar sweetened beverages and unhealthy snacks	• Taxation will negatively affect the population because sweet drinks and sweets give people pleasure • The industry lobbyists, the Ministry of Finance and Parliament members will oppose.
Potential supporters of taxation	• The Government, the Ministry of Finance • Parliament members • The Ministry of Education, education organizations • Parents • Health maintenance organizations, the Ministry of Health, health promoting organizations, health professionals (physicians, nutritionists). • Environmental organizations
Reference to low socioeconomic populations	• Taxing cause inequality because it affects the poor much more than the rich.
Alternative strategies to fight obesity	• Regulation and responsibility of the industry • Build a healthier environment • More physical activity • Personal education and responsibility
Tax rate and its effect on consumption	• Low taxation does not make any consumers change • High taxation is not proportionate
Public response to the taxation	• The public will oppose, they will never support additional taxation (it is not a popular move) • The public will be indifferent
Barriers and obstacles to implementing the tax	• Difficulties in defining healthy and unhealthy products • Difficulties in deciding which products to tax. • Higher prices of healthy food • Difficulties in passing the message to the public • Technical and logistic problems with taxation • Enforcement difficulties • Damage to the Industry and danger of worker termination
Tax complementary activities	• Taxing needs a supporting educational and public promotion and advocacy to follow
The need for earmarking tax revenue and obstacles to implementation	• The tax revenue will not be used for the tax cause. • Government authorities will oppose. • Learning from the past- the attempt to use the tobacco tax revenue for health cause was a failure. • Opinions against the use of the tax revenue for a specific cause. • The public will support the use of tax revenue for health cause, and there will be less objections to the tax. • Options for the revenue use: health promotion, health education, subsidies for healthy foods.

### Responsibility for obesity and its prevention

All of the interviewees, regardless of sector, regarded obesity as a combined public and personal problem.

*“Obesity is a disease; it is defined today as a disease. And it is more than a private problem of the individual; it’s a typical phenomenon of a consumer society*”. (Legislator 13).

*“I think it’s both. There is the individual responsibility that he cannot ignore. And there is the societal responsibility*”. (Media 37).

Two of the interviewees regarded obesity primarily as a personal problem, but acknowledged that the implications of obesity extend beyond affected individuals to impact society as a whole.

Most interviewees thought that prevention of obesity should be under public responsibility due to the economic burden it confers on society as a whole:

*“It has a long-term effect on the budget… not everybody understands the concept that obesity has long-term social and economic effects, it is a burden on the healthcare system*”. (Legislator 16).

“*Obesity is a public concern- it’s a burden on the national budget*”. (Industry 28).

### Views on taxation

#### Prevention of obesity by taxation of SSBs and unhealthy snacks

Interviewees from the economics sectors were the only ones who hypothesized that a tax on unhealthy foods and drinks would definitely reduce the consumption of these products.

“*Yes definitely, tax is a very important tool for changing behavioral patterns. I am definitely pro taxation*”. (Economists 33).

The prevailing notion among non-economists was that increasing the prices of SSBs and unhealthy snacks would not have a substantial effect on reducing consumption among the general population.

“*It won’t work, if you ask me it won’t work*”. (Food and beverages industry 27).

“*I don’t think it will work. No, it won’t work, here it won’t work*”. (Public Representatives 9).

However, some of the interviewees suggested that such taxation would only affect individuals and families from lower socioeconomic backgrounds, while the rest of the population would remain indifferent to the price increase.

The interviewees suggested that potential supporters of taxation would be government offices such as the Ministries of Finance, Education and Health, Members of Parliament, as well as educational, health and environmental organizations, health professionals and parents.

*“I think that if it will cost more they [the parents] won’t have a choice and they won’t buy it. They will argue with their children and they won’t buy it*” (Health professions 21).

Interviewees most commonly identified the food and beverage industry as the main objector to the initiative of taxation on SSBs and unhealthy snacks.

“*I think that manufacturers of soda and chocolate and those kinds of food will object because its will decrease their income*”. (Legislator 14).

Other potential objectors identified by interviewees were members of the tax system: the Ministry of Finance and the Israeli Tax Authority, as well as Members of Parliament and liberal organizations.

#### Positions against taxation

Several arguments against taxation were raised. One of the arguments was that such action would be deemed as a paternalistic move that would impede the freedom of the individual:

“*There should be a limit to government intervention, even if the cause is just and for good values. I think there should be limits to what the government is allowed to intervene in*.” (Legislators 16).

Almost all of the industry representatives as well as a few of the regulators, health professionals and media representatives mentioned that snacks and soft drinks are a source of pleasure and that taxing them would harm the public:

“*We also eat for our soul*”. (Regulators 3).

“*People today like to drink sweet drinks, like to eat candy; it helps them relieve their stress*”. (Health professions 19).

Some of the stakeholders said that the overall tax burden in Israel is already heavy and using tax, even if for the purpose of health promotion, can negatively affect the public.

“*In the last few years there is a big increase in overall taxes so it won’t be received as a positive move*”. (Media 37).

“*The taxes are very high and I don’t want them to be higher, not even for important causes*” (Legislators 16).

However, some stakeholders thought that a low tax rate would not make any difference because it would not have an influence on consumption of these products.

Interviewees from all sectors, except for those from the industry and the economists, repeated the theme that this type of tax is regressive and would therefore selectively affect the lower socioeconomic echelons of society:

“*The food expenses in the lower socio economic populations take a larger percentage of their budget so it would negatively affect them more*”. (Health professions 24).

“*It reduces the amount of money available to lower income families to spend on necessary items*”. (Legislators 14).

“*The problem is more serious in the poorer populations*”. (Public Representatives 9).

*“Raising the prices would hurt the ones who don’t have money*”. (Public Representatives 10).

“*It will influence the weaker crowds,* [the ones] *it can influence*”. (Media 38).

Although the interviewees pointed to the disproportionate adverse effect of the tax on low socioeconomic populations, some of them also indicated that lower socioeconomic population groups consume more “unhealthy” foods and drinks. As a part of their culture:

“*Usually, lower socio-economic populations consume less healthy food products*”. (Economists 33).

“*The ultra-orthodox always buy these drinks on the weekends*”. (Regulators 8).

“*The Ethiopians adopted Coca Cola because it is similar to their traditional drink*”. (Regulators 3).

#### Barriers and challenges to implementation of taxation

Several potential obstacles and challenges to implementation of taxation were mentioned. The stakeholders thought that taxation would not convey a positive message or the right message to the public:

“*The challenge is to deal with the public, how to create a program that won’t cause objections*”. (Media 37).

*“I think that the biggest challenge would be how to convey this message to the public, as something for their own benefit.”* (Health Professions 20).

Additionally, there would be technical and bureaucratic obstacles related to difficulty of legislation and enforcement of taxation.

“*The first problem is enforcement which is always a problem in Israel for some reason*”. (Health Professions 18).

“*The question is how you collect this kind of tax; it requires more bureaucracy and more mess”*. (Food and beverages industry 27).

Furthermore, it would be difficult to define the products that should be taxed because there is no clear definition for which foods account as “unhealthy” ….

“*The definition of healthy is controversial, healthy is not just what is in the food, it is also a matter of quantities. There are unhealthy foods but there are some which are in-between*”. (Health Professions 23).

### Possibilities of development of a “black market” and attempts to avoid the tax were mentioned

“*The tax will increase the price and if there is a demand for the product what’s going to happen is the development of a black market or another thing that can happen is that people would deceive the tax authorities by selling the taxed products without the tax*”. (Regulators 5).

The stakeholders thought that in an attempt to prevent taxation, manufactures would threaten that they would have to close down factories due to reduced sales and therefore let go of workers, leading to unemployment.

“*The strongest objection would be the one mounted by manufactures. They won’t hesitate to use the argument of closing factories and firing employees*”. (Public Representatives 9).

The higher price of healthy foods was also noted as an obstacle to implementation of taxation as consumers would not be able to afford healthier options:

“*The first and most problematic obstacle is the one I mentioned, that even with the taxation, these products are going to be less expensive than the healthy products which are too expensive*”. (Media 37).

#### Complementary and alternative strategies to taxation

Stakeholders from all the sectors but the industry agreed that taxation should not be the only measure taken for preventing obesity; rather it should be accompanied by educational and public support. However, health professionals thought that education should be one component of the strategy to decrease consumption of unhealthy foods while interviewees from the industry and the media thought that education is the only strategy needed.

*“I am siding with the tax. But I think that by itself it won’t be enough, the prevention proportions would be low”*. (Health Professions 20).

*“It can’t be a “one time” and only one measure. It must come with complementary measures such as a public-relations campaign and education”*. (Economists 33).

“*The only thing that will work is education; to deal with it* [the obesity problem] *it’s just education. And it should start from kindergarten*”. (Food and beverages industry 27).

“*First of all and the most important solution is education and there I would invest first, before all other things*”. (Media 38).

Additional strategies that were suggested for fighting obesity were regulation and industry responsibility, building healthier environments, encouraging more physical activity and personal responsibility.

One of the options for complementary measures to taxation is to use the tax revenue for health purposes. Interviewees assessed that such pre-allocation may contribute to enhancement of support in favor of the tax in some sectors and would receive support of the public. However, they raised the concern that the tax infrastructure in Israel would not permit earmarking the tax revenue for health promotion purposes and suggested that government authorities and other sectors may oppose it. It was mentioned that a past attempt to use the revenue from the tax on tobacco for a health cause had failed.

“*The ministry of finance is against using any tax revenue for specific purposes; they will put tax on snacks and use the money to appoint 30 new ministers*.” (Regulators 6).

*“We know that the Ministry of Finance does not like the use of tax money for a specific cause. The Ministry of Finance wants every penny they collect to be free for use towards the purposes that they choose”*. (Legislators 14).

*“We don’t live in an ideal world; the revenue from the tax on cigarettes doesn’t go to the ministry of health”*. (Regulators 8).

*“I am strongly against funding moves with more taxes”.* (Legislators 16).

“*Definitely, the tax revenue must be dedicated to this issue*”. (Media 36).

“*I think it would be more effective and the public would accept it better”*. (Economists 33).

When asked which purposes the tax revenue should fund, three main purposes came up: health education, health promotion and healthy food subsidies.

*“If the government says we took 100 million NIS here and subsidized 100 million in diet food, it seems fair to me*”. (Food and beverages industry 27).

“*It should not just provide a negative incentive to buying the unhealthy foods, it also gives a positive incentive to buy the healthy ones*”. (Policy makers 1).

*“If you take this money for a specific purpose it must be fighting obesity”*. (Legislators 16).

*“I would use it for parks, walking and biking trails”*. (Regulators 3).

*“I think the money needs to go to education”*. (Health Professions 26).

#### Public response to taxation

When asked about the anticipated public response to taxation, some of the stakeholders said that the public would oppose such taxation while others said that the public would be indifferent.

*“I don’t think the public would stand up and shout”*. (Health Professions 26).

*“Most [of the public] would oppose”.* (Media 38).

As mentioned above, the interviewees assessed that pre-allocation of the tax revenue toward health promotion purposes may contribute to enhanced public support.

## Discussion

This study has revealed that there is a cross-sector perception among stakeholders that the responsibility for obesity prevention should be shared between individuals and society. This finding is in agreement with the view of the World Health Organization that a full effect on individual responsibility can only occur if individuals (especially poorer ones) have access to an affordable healthy lifestyle supported through sustained political commitment and the collaboration of many public and private stakeholders [3].

The World Health Organization has suggested using economic tools, such as the use of targeted taxes and/or subsidies to discourage the consumption of less healthy options and to improve the consumption of healthier food products by increasing accessibility, availability and affordability [30, 31]. In the current study only stakeholders from economic sectors thought that taxation would reduce consumption of SSBs and unhealthy foods, while the prevailing notion among non-economists was that such a tax would not be acceptable because the higher price would not decrease consumption. Contrary to this view, recent reports from countries where a tax on SSBs was implemented show otherwise. In France, there was a decrease of 6.7% in demand for regular cola in the first 2 years after an 11 euro-cent per 1.5-l SSB excise tax. In Mexico, following the implementation of a 1 peso per liter excise tax to SSBs and an 8% tax on nonessential energy-dense food, purchases of taxed beverages decreased by 5.5% in the first year and by 9.7% in the second year, yielding an average reduction of 7.6% over the first 2 years of implementation. Purchases of untaxed beverage increased by 2.1% and water purchases also increased [12]. Consumption of SSBs in the US city of Berkeley, California, fell after the city imposed a “penny per ounce” tax on SSBs (sugar sweetened sodas, fruit flavored drinks, sweetened water, coffee, tea products, and syrups used in making SSBs) while water consumption rose [32, 33].

A major concern that was raised was the regressive nature of the tax and its effect on lower socioeconomic populations. In Mexico the magnitude of changes in SSB consumption was greater in lower-income and urban households [11, 12]. In low-income neighborhoods in Berkeley, SSB consumption declined by 21% over a 1-year period from before the tax to after the tax, and increased by 4% in the comparison neighborhoods over the same period [32, 33].

Some of the stakeholders viewed the proposed tax as a paternalistic move that would impede the freedom of the individual, and would add to the tax burden. However, it was suggested that the costs of obesity arising from individuals’ poor nutritional choices are borne by society as a whole through taxes, lost productivity, and an overburdened health care system [34]. Most of the stakeholders agreed that taxation should be only one aspect of the obesity prevention strategy and mentioned that it should be accompanied by educational support. In a study that assessed the opinions of 21 Spanish stakeholders from food and physical exercise policy networks on public policy options for responding to obesity, the more popular policy options were educational initiatives including: the inclusion of food and health education in the school curriculum, improving health education to the general public, improving the training of health professionals in obesity care and prevention and incentives to caterers to provide healthier menus and improve community sports facilities, while fiscal measures as subsidies and taxes had the lowest support [35]. In the United Kingdom, the introduction of a £0.10 levy on SSBs in a restaurant chain that was accompanied by supporting activity including beverage menu redesign, new products and establishment of a children’s health fund from levy proceeds, was associated with declines in SSB sales per customer in the short and medium term, particularly in restaurants with higher baseline sales of SSBs [36]. Pomeranz has suggested that this is also true for the case of tobacco taxation whereby excise taxes alone were not responsible for reducing tobacco consumption, but rather a combination of strategies, including indoor smoking bans, marketing limits and price increases, worked together to decrease cigarette use [37].

Some of the stakeholders interviewed in our study said that the public would oppose such taxation while others said that the public would be indifferent. Only parents were mentioned as possible supporters of the tax. This is contradictory to the findings of an Australian Citizens’ Jury that dealt with the question of whether taxation on food and drinks is an acceptable strategy to the public in order to reduce rates of childhood obesity. The jury unanimously supported taxation on sugar-sweetened drinks but generally did not support taxation on processed meats, snack foods and foods eaten or purchased outside the home. The Jury further suggested that the general public might support taxation on sugar-sweetened drinks to reduce rates of obesity in children [38]. The French sugar-sweetened beverage tax appeared to be favorably perceived by the public, with people with lower education more supportive than those with higher education; 57.7% perceived it as helpful in improving population health. Participants were more likely to support the tax model if the revenue it generated would be used for health-care system improvement (72.7%) and if such taxation was associated with a corresponding decrease in the prices of other foodstuffs (71.5%) [39]. Indeed, our interviewees assessed that pre-allocation of the tax revenue toward health promotion purposes may contribute to enhancement of support of the public. However, one of the lessons learned from implementing sugar taxes in the Pacific islands, was that while the earmarking of taxes for health had been widely recommended, the revenue could be redirected as government priorities changed [15]. In Mexico, the tax revenue had not been specifically earmarked, but the senate made a resolution to use part of the taxes for providing potable water to public schools, particularly in low-income areas [11]. Revenues from the SSB tax implemented in Berkeley, California are used for supporting municipal health and nutrition programs [40]. A different approach for gaining support for a tax on SSBs was taken in Philadelphia where, in order to shift claims on government involvement in individual behavior, the Philadelphia SSB tax proposal was introduced with the explicit goal of financing universal prekindergarten, a goal for which broad support existed, and deliberately not framed as a health intervention [41]. In contrast, politicians in Denmark considered the ‘fat tax’ as a funding source rather than a public health initiative, which resulted in its significant shortcomings. It received criticism for being poorly designed and gradually lost popularity among health professionals, politicians and the public [42]. A systematic review of the research on health taxes indicated that policymakers should be clear about the primary goal of any health tax and frame the tax accordingly, as not doing so leaves taxes vulnerable to hostile lobbying. Conversely, earmarking health taxes for health spending tends to increase public support so long as policymakers follow through on specified spending commitments [43].

Health-related taxes and subsidies on food challenge the interests of the food industry whose profit motive can be in direct conflict with public health goals [44]. An example was seen in Denmark where the industry and trade associations were heavily involved in the political process of formulating the ‘fat tax’ on butter, milk, cheese, pizza, meat, oil and processed food if the item contained more than 2.3% saturated fat. Industry representatives used certain tactics to oppose the ‘fat tax’: threatening lawsuits, predicting welfare losses, casting doubt on evidence, diverting focus and requesting postponement. After the ‘fat tax’ was implemented, the food industry continued their opposition through intensified lobbyism and juridical actions at EU level. However, other factors seem to have contributed to the fall of the ‘fat tax’ [42]. Analysis of four different soft drink taxes in Pacific countries (Fiji, Samoa, Nauru and French Polynesia) revealed an interaction between the Ministries of Health, Finance and Revenue at every stage of the policy making process. Relevance to government fiscal priorities was important in gaining support for soft drink taxes. The active involvement of health policy makers was also important in initiating the policies, and the use of existing taxation mechanisms enabled successful policy implementation [15]. Some of the stakeholders thought that in an attempt to prevent taxation, manufactures would threaten that they would have to close down factories due to reduced sales and therefore let go of workers, leading to unemployment; however this concern may not realize itself according to a study that assessed changes in employment in the manufacturing industry, the commercial sector and national unemployment rates, associated with the fiscal policies implemented in Mexico. The study’s results showed that there were no employment reductions associated with these policies [45].

A recently-published study on the positions of 20 New Zealand stakeholders concerning the feasibility and acceptability of selected health-related food taxes and subsidies over the next 5 to 10 years, showed similar arguments to those presented by our interviewees for and against such taxes. However, contrary to our findings, the stakeholders in New Zealand viewed the soft drink tax as likely acceptable to the public and there did not appear to be any major issues in the New Zealand context that would impact the feasibility of implementing such tax. Support for a subsidy on fruit and vegetables was also quite strong while there was little support for a tax on saturated fat or salt [46].

The views represented in this study are specific to its 39 participants. Although their positions as leaders in their fields make them appropriate representatives of their sectors and the type of reactions they may have to a taxation policy on SSBs and unhealthy snacks, given the qualitative nature of the study, their views may not be representative of all stakeholders in their sector. In addition, the views expressed in the interviews may have been biased by social desirability. We attempted to limit such bias by explaining to the participant prior to the interview that participation in the study was done under his/her professional role; however, participants may have expressed their own personal opinions in order to please the study team. Interpretation by reflection of responses was conducted by the authors through their professional and own opinions, but the consistency of many of the findings with those from other sources lends validity.

Our study is unique in that it presents the views of stakeholders from all of the sectors involved in making of this policy, and shows barriers that may be expected when implementing it. Although some time has passed since data collection, this issue is still very much relevant as shown in the discussions of the regulatory committee established by the Israel Ministry of Health in 2016. This committee aimed at regulating food quality in order to provide healthier food choices. Among the issues discussed were restricting advertising and marking of unhealthy food products. Although the issue of taxation of such food products was raised several times and discussed extensively, the idea was rejected due to various coalition considerations and the unwillingness of the Health and Finance Ministers to impose additional taxes on the population. It was decided to first follow other regulatory measures and examine their effects, and only then to consider taxation. In December 2017, the Israeli Parliament’s Health, Labor and Welfare Committee approved regulations, which will be in effect as of 2020, requiring foods deemed to have unhealthy levels of sugar, sodium or saturated fat to have red warning labels on them. These regulations were intensely lobbied against by the food industry, which promoted a voluntary program for labeling instead of one mandated by the government. Another measure taken to reduce the consumption of unhealthy food items was the Ministry of Health’s decision to remove products such as SSBs and high-sodium food products from school vending machines and kiosks.

## Conclusions and policy recommendations

Our in-depth interviews have revealed that leaders from all sectors acknowledge the societal responsibility for reducing the rates of obesity. They all agree that there is a need for policies that will assist individuals, especially the under-privileged, in making better food choices. A fiscal policy to tax SSBs and unhealthy snacks may help achieve that but it must be supplemented by other policy measures and norms. Such a policy may be better accepted by health policy makers if the tax revenue is earmarked to fund healthier choices including subsidies of healthy foods and health promotion activities and comprise one of the major steps for a healthier nation.

While the perspective of the stakeholder groups explored here provide valuable insights, additional research (e.g., public opinion, estimates of price elasticity to predict changes in consumption rates) is needed to inform these questions in the Israeli context.
